# NOTCH3 as a prognostic biomarker and its correlation with immune infiltration in gastrointestinal cancers

**DOI:** 10.1038/s41598-024-65036-x

**Published:** 2024-06-21

**Authors:** Jia Xu, Xiao-li Jin, Hao Shen, Xuan-wei Chen, Jin Chen, Hui Huang, Bin Xu, Jian Xu

**Affiliations:** 1 School of Medical Technology and Information Engineering, Zhejiang Chinese Medical University, Hangzhou, 310053 Zhejiang People’s Republic of China; 2 Department of General Surgery, Sir Run Run Shaw Hospital, School of Medicine, Zhejiang University, Hangzhou, 310016 Zhejiang People’s Republic of China

**Keywords:** NOTCH3, Gastrointestinal cancers, Tumor infiltration, Immune, Gastrointestinal cancer, Cancer, Cancer, Gastroenterology, Oncology

## Abstract

NOTCH receptor 3 (NOTCH3) is known to regulate the transcription of oncogenes or tumor suppressor genes, thereby playing a crucial role in tumor development, invasion, maintenance, and chemotherapy resistance. However, the specific mechanism of how NOTCH3 drives immune infiltration in gastrointestinal cancer remains uncertain. The expression of NOTCH3 was analyzed through Western blot, PCR, Oncomine database, and the Tumor Immune Estimation Resource (TIMER) site. Kaplan–Meier plotter, PrognoScan database, and gene expression profile interactive analysis (GEPIA) were used to assess the impact of NOTCH3 on clinical prognosis. The correlation between NOTCH3 expression and immune infiltration gene markers was investigated using TIMER and GEPIA. NOTCH3 was found to be commonly overexpressed in various types of gastrointestinal tumors and was significantly associated with poor prognosis. Furthermore, the expression level of NOTCH3 showed a significant correlation with the tumor purity of gastrointestinal tumors and the extent of immune infiltration by different immune cells. Our findings suggest that NOTCH3 may act as a crucial regulator of tumor immune cell infiltration and can serve as a valuable prognostic biomarker in gastrointestinal cancers.

## Introduction

Gastrointestinal tumors are a serious threat to people’s lives and health. Epidemiological studies^1^ show that the incidence of colorectal cancer ranks first in the world, the incidence of gastric cancer is the fifth, the mortality rate is the third^2^, and the incidence of liver cancer is 4.7%^3^. Ranked sixth in gastrointestinal tumors, pancreatic cancer is a globally recognized malignant tumor with a very poor prognosis, with a one-year survival rate of less than 40% after diagnosis^4^. Cancer metastasis is an important reason for the high mortality rate. Tumors effectively suppress the immune response by activating negative regulatory pathways related to immune homeostasis (also called checkpoints) or by adopting features that enable them to actively evade detection^5–7^.

Immune-related mechanisms, such as cytotoxic T lymphocyte associated antigen 4 (CTLA4), programmed death-1 (PD-1) and programmed death ligand-1 (PD-L1) inhibitors, play an important role in tumors. In melanoma, small cell lung cancer, and kidney cancer, specific PD-1 and PD-L1 immune checkpoint inhibitors (anti-PD-L1/PD-1) have shown therapeutic effects beyond traditional radiotherapy and chemotherapy^8^. Surprisingly, some tumor patients who have received multiple standard treatment failures have sustained relief of symptoms and a significant increase in survival time after treatment with anti-PD-L1 and PD-1^9^. In recent years, tumor immunotherapy has overturned the traditional tumor treatment methods based on surgery, radiotherapy and chemotherapy^10^. Ewa M Nowosielska^11^ found that whole-body irradiations (WBI) alone and in combination inhibits cytotoxic T lymphocyte antigen 4 (CTLA-4) and programmed death 1 (PD-1) receptor immune checkpoint (IC) and/or heat shock protein 90 (HSP90) significantly reduced mouse lung tumors, and inhibited the cloning potential of Lewis lung cancer (LLC1) cells in vitro.As reported that the tumor of a single colorectal cancer patient that response to PD-1 blockade has a mismatch repair defect^12^. Therefore, it is reasonable to believe that tumors deficient in mismatch repair are more sensitive to PD-1 blockade than tumors deficient in mismatch repair^13^.

NOTCH signaling is highly conserved in multicellular organisms, and it is involved in cell fate decision, cell proliferation/differentiation and lineage specification^14,15^. In mammals, there are four NOTCH receptors (NOTCH1-4) and five ligands [Jagged (JAG)1, 2 and Delta-like ligand (DLL)1, 3 and 4], which participate in the activation of NOTCH signal. A previous study further found^16^ that the overexpression of NOTCH3 prevents the cell cycle in G0/G1 phase. Overexpressing the intracellular domain of NOTCH3 (N3ICD) can up-regulate the expression of Cdh1 and cause p27Kip accumulate by accelerating the degradation of Skp2, as well as inhibit the breast cancer cell line, MDA-MB-231, proliferation and colony formation rate. That’s to say NOTCH3 may arrest cell cycle progression of MDA-MB-231 by regulating the Cdh1/Skp2/p27kip1 signaling axis. Wang^17^ found that miR-206 can inhibited CRC cancer cell proliferation and migration, blocked the cell cycle, and activated apoptosis, all of which are related to the NOTCH3 signaling pathway. The tumor suppressive ability of miR-206 may be explained by directly inhibiting the NOTCH3 signaling pathway and indirectly interacting with other signaling pathways involving CDH2 and MMP-9^18^.

The transcription targets regulated by NOTCH may be an oncogene or tumor suppressor gene; however, NOTCH3 is a potential oncogene. There have been many reports in the literature that NOTCH3 signaling may play an important role in tumor development, invasion, maintenance, and chemotherapy resistance^16,19,20^. However, the potential functions and mechanisms of NOTCH3 in tumorigenesis and tumor immunology are still unclear. Therefore, there is an urgent need for the elucidation of the immunophenotypes of tumor-immune interactions and identification of novel immune-related therapeutic targets in cancers.

In this study, we conducted a comprehensive assessment of the relationship between NOTCH3 and the prognosis of cancer patient using databases including Oncomine, PrognoScan and Kaplan–Meier plotter. Besides, we further investigated the connection of NOTCH3 and immune cell infiltration in tumors by the Tumor Immunoassay Resource (TIMER). The results show that novel insights in the functional role of NOTCH3 in gastric cancer, thereby highlighting a potential mechanistic basis whereby NOTCH3 influences immune cell interaction in tumors.

## Materials and methods

### Cell culture and treatment

The School of Medical Technology and Information Engineering at Zhejiang Chinese Medical University (Zhejiang, China) provided the stomach tissue of normal rats and the human gastric cancer cell line HGC-27. 10% fetal bovine serum (FBS; Biological Industries, Kibbutz Beit Haemek, Israel) was added to the RPMI-1640 medium (Hyclone; GE Healthcare Life Sciences, Logan, UT, USA) in which the cells were cultured. Every cell was cultured at 5% CO_2_, saturated humidity, and 37 °C.

### RNA isolation and quantitative real‑time PCR (qRT‑PCR)

Trizol was used to extract total RNA from samples of cells and tissue. Following the manufacturer’s instructions, reverse transcription was carried out using the Takara Reverse Transcription Kit, and real-time quantitative PCR was carried out using the Takara SYBR Premix ExTaq kit. In Table 1, the primers are displayed.

**Table 1:** The sequences of primers.

Name	Sequences
GAPDH	F: 5ʹ-ACCACAGTCCATGCCATCAC-3ʹ
	R: 5ʹ-TCCACCACCCTGTTGCTGT-3ʹ
NOTCH3	F: 5ʹ-GTCGTGGCTACACTGGACCT-3ʹ
	R: 5ʹ-AATGTCCACCTCGCAATAGG-3

### Western blot analysis

Protein loading buffer (Beijing Solarbio Science & Technology Co., Ltd., Beijing, China) containing 1% PMSF protease inhibitors was used to collect tissue and lyse HGC-27 cells. The tissue and cells were first lysed for ten minutes on ice, after which they were moved to centrifuge tubes and boiled to 100 °C for ten minutes in boiling water. A BCA Protein assay kit (Pierce; Thermo Fisher Scientific, Inc.) was used to quantify the total protein concentration in accordance with the manufacturer’s instructions. Ten microliters of protein per lane were added and separated for one hour at room temperature on an 8% SDS-PAGE. Membranes made of polyvinylidene difluoride were used to receive the resolved proteins. The membranes were transferred in cold transfer buffer for two hours, blocked with 5% skim milk for two hours at room temperature, and then incubated with primary antibodies [Notch3, NLRP3, COX-2, NF-κB p65, NF-κB p-p65, GAPDH] for an entire night at 4 °C. After that, membranes were incubated for two hours at room temperature with secondary horseradish peroxidase (HRP)-labeled antibodies (goat anti-rabbit IgG). The ideal exposure blot was then chosen using Image LabTM software 4.1 (Bio-Rad Laboratories, Inc., Hercules, CA, USA) and Electro-Chemi-Luminescence detection reagent (Thermo Fisher Scientific, Inc.). Densitometric analysis using ImageJ Software 1.4.3.67 (National Institutes of Health, Bethesda, MD, USA) was used to detect changes in protein abundance. The results were normalized to GAPDH, as previously described.

### Antibodies

The commercial primary antibodies utilized in this investigation are anti-GAPDH (Affinity, AF7021), anti-NLRP3 (abcam, ab263899), anti-COX-2 (Cell Signaling Technology, #4842), anti-NF-κB p65 (Beyotime, AF0246), anti-NF-κB p-p65 (Beyotime, AF5875) and anti-Notch3 (abclonal, A13522). The following secondary antibodies were employed in this investigation: Goat anti-rabbit IgG tagged with HRP (Sangon Biotech, D111018).

### Ocomine database analysis

Using the extensive and robust online cancer database Ocomine (http://www.oncomine.org), we were able to identify the NOTCH3 DNA and mRNA expression levels in a variety of tumor types. The following describes how the filter condition results were set: Fold change > 1.5, *P*-value < 0.001, and gene ranking overall.

### PrognoScan database analysis

Using a sizable collection of publically accessible microarray datasets, the PrognoScan database (http://www.abren.net/PrognoScan) was utilized to determine the relationship between NOTCH3 expression and prognosis in a variety of malignancies. The Cox *P*-value < 0.05 was used to recalculate the cutoff.

### Kaplan–Meier plotter database analysis

Using the Kaplan–Meier plotter (http://kmplot.com/analysis/), the relationship between NOTCH3 expression and survival in gastric, ovarian, lung, and breast cancers was examined. Additionally, the log-rank *P*-value and the hazard ratio (HR) with 95% confidence intervals were calculated.

### TIMER database analysis

TIMER (https://cistrome.shinyapps.io/timer/) is a comprehensive resource for systematic investigation of immune infiltration of various cancer types. It estimates the abundance of immune infiltration using 10,897 samples of 32 cancer types from the Cancer Genome Atlas (TCGA). The expression of NOTCH3 in various cancer types and the relationship between NOTCH3 expression and the quantity of immune infiltrate—which includes B cells, CD4 + T cells, CD8 + T cells, neutrophils, macrophages, and dendritic cells—were examined in the TIMER database database.

### Gene correlation analysis in GEPIA

RNA-seq data from 9736 tumour samples and 8587 normal control samples from the TCGA and GTEx data sets may be analyzed uniformly using the online database GEPIA (http://gepia.cancer-pku.cn/index.html). The GEPIA database evaluated the expression of specific markers linked to immune cell infiltration as well as the relationship between NOTCH3 expression and patient prognosis in a variety of tumour types. Other genes of interest were displayed on the y-axis, while NOTCH3 was utilized for the x-axis.

### Statistical analysis

For the purpose of creating survival plots in their respective analyses, the PrognoScan, Kaplan–Meier plotter, TIMER, and GEPIA databases were shown. The data included either HR and P-values or P-values obtained from a log-rank test. The NOTCH3 expression’s correlation was assessed using Spearman’s correlation and statistical significance. The correlation’s strength was determined by calculating the r values, which ranged from 0.00 to 0.19 for “very weak,” 0.20–0.39 for “weak,” 0.40–0.59 for “moderate,” 0.60–0.79 for “strong,” and 0.80–1.0 for “very strong.” A *P*-value of less than 0.05 was deemed statistically significant.

## Results

### NOTCH3 expression is signifcantly upregulated in gastric cancer

**Figure 1:**
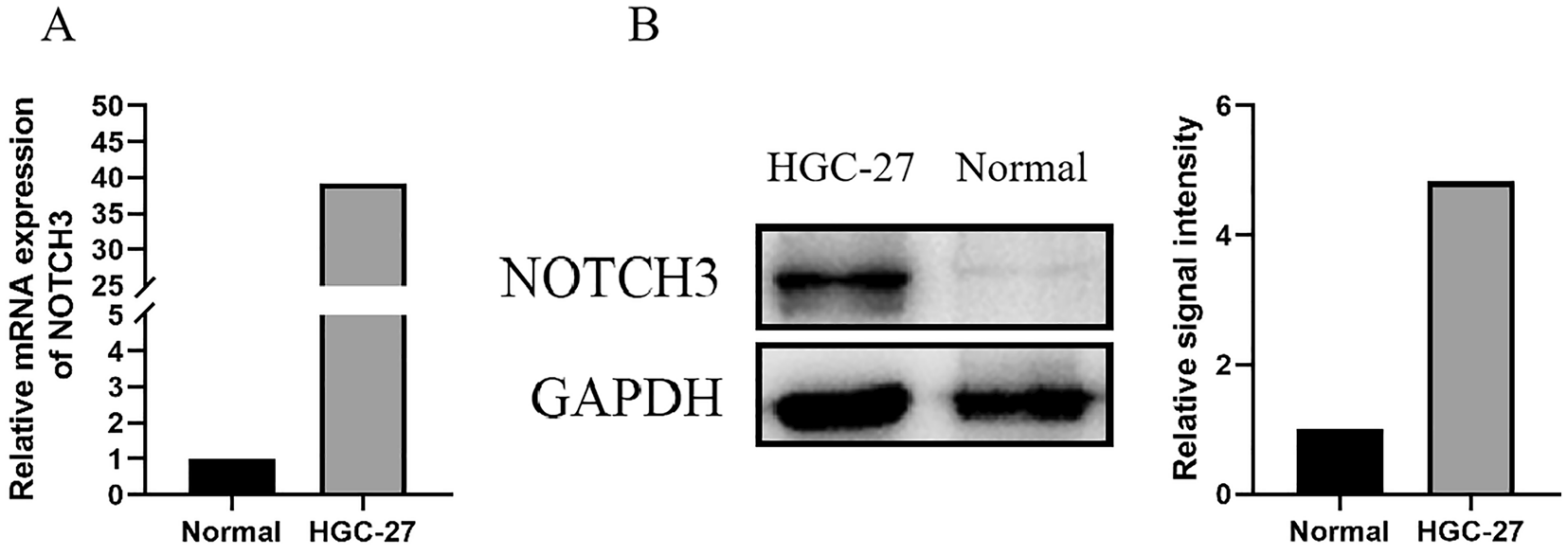
NOTCH3 mRNA and protein expression in the gastric cancer cell. (**A**) Relative NOTCH3 gene expression was examined in the gastric cancer cell line and the stomach tissue of normal rats by real-time PCR. (**B**) Expression of NOTCH3 protein in the indicated cell lines was determined by western blotting.

Using real-time PCR and western blot analysis, we first examined NOTCH3 mRNA and protein expression in the stomach tissue of normal rats and the human gastric cancer cell line HGC-27 to ascertain if NOTCH3 is involved in the formation of gastric cancer. Figure 1A and B show that NOTCH3 mRNA and protein expression were found in gastric cancer cell line, but not in normal gastric tissue. When combined, our findings demonstrated that gastric cancer cells have significantly elevated NOTCH3 mRNA and protein expression.

### The DNA and mRNA expression levels of NOTCH3 in various types of human cancers

**Figure 2:**
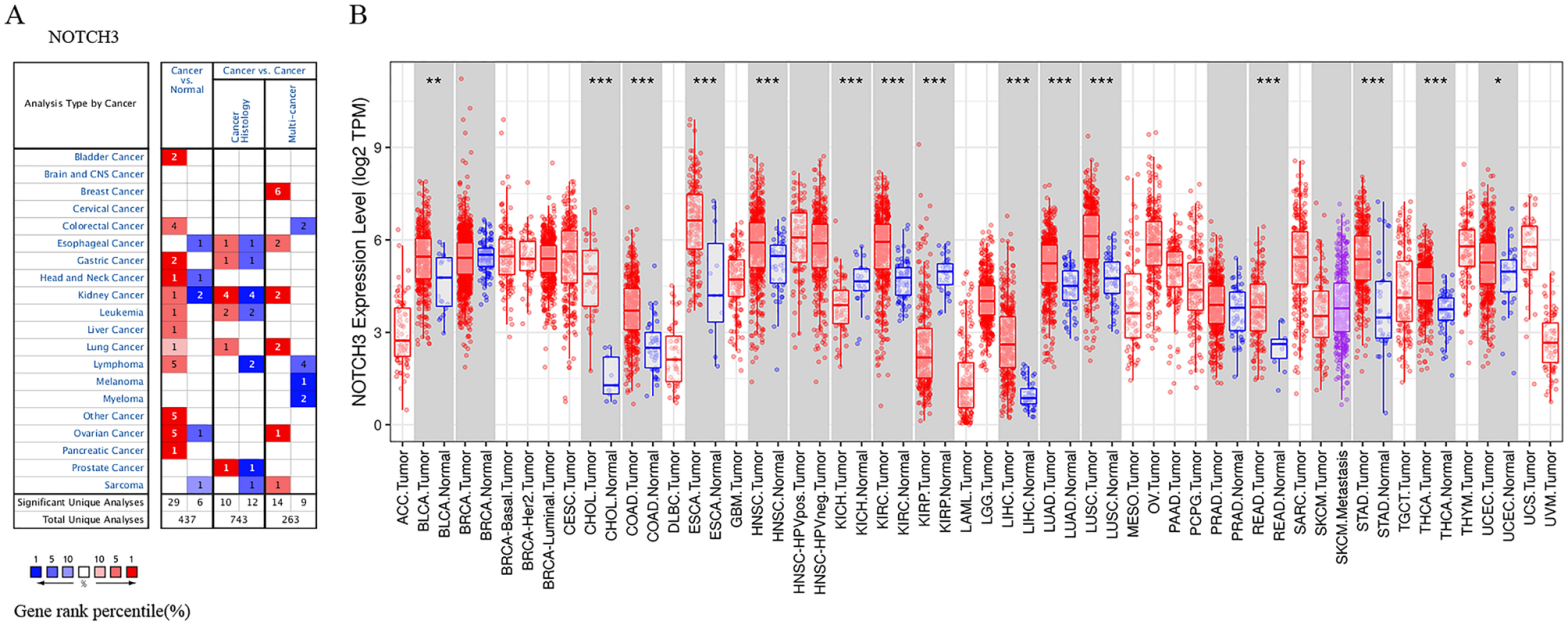
The expression level of NOTCH3 in different types of human tissues. (**A**) Upregulated or downregulated NOTCH3 in different human cancers in the Oncomine database. We set the filter condition as: *p* value < 0.0001, fold change > 2, gene rank: 10%, data type: All. (**B**) Human NOTCH3 expression levels between tumors and normal tissues in TIMER database. (*P < 0.05, **P < 0.01, ***P < 0.001).

The Ocomine^21^ database provided a pan-cancer investigation of NOTCH3 expression levels, revealing differential NOTCH3 expression in various human tumours. The findings showed that, in comparison to normal tissues, NOTCH3 expression was lower in sarcoma, esophageal, head and neck, kidney, leukemia, liver, lung, pancreatic, and colorectal cancers but higher in bladder, colorectal, gastric, head and neck, kidney, and leukemia cancers (Fig. 2A).Remarkably, some data also showed that NOTCH3 was downregulated in sarcoma, renal, ovarian, esophageal, and head and neck cancers. Based on all TCGA tumours, we discovered that NOTCH3 was significantly higher in BLCA (bladder urothelial carcinoma), CHOL (cholangiocarcinoma), COAD (colon adenocarcinoma), ESCA (esophageal carcinoma), HNSC (head and neck cancer), KIRC (kidney renal clear cell carcinoma), LIHC (liver hepatocellular carcinoma), LUAD (lung adenocarcinoma), LUSC (lung squamous cell carcinoma), READ (rectum adenocarcinoma), STAD (stomach adenocarcinoma), THCA (thyroid carcinoma), and UCEC (uterine corpus endometrial carcinoma) in comparison to normal controls. On the other hand, NOTCH3 expression was downregulated in kidney renal papillary cell carcinoma (KIRP) and kidney chromophobe (KICH) as compared to normal tissues. To gain a deeper understanding of the correlation between NOTCH3 expression levels and clinicopathological characteristics in gastrointestinal malignancies, the Kaplan–Meier Plotter database was utilized. Gender, differentiation, and Lauren categorization (*P* < 0.05) in Table 1 indicated a strong correlation between NOTCH3 overexpression and declining OS and PFS (Table 2).

**Table 2:** Correlation of NOTCH3 and clinicopathological factors in STAD from Kaplan–Meier Plotter database.

Clinicopathological	Overall survival (n = 875)			Progression-free survival (n = 640)		
characteristics	N	Hazard ratio	*P*-value	N	Hazard ratio	*P*-value
sex						
Female	236	3.62(2.11–6.21)	6.30e−07	201	3.14(1.82–5.43)	1.40e−05
Male	544	2.64(2.09–3.34)	< 1e−16	437	2.56(2–3.27)	1.00e−14
Stage						
1	67	7.38(0.96–56.63)	0.024	60	4.75(0.6–37.57)	0.1
2	140	2.09(1.13–3.88)	0.017	131	1.72(0.94–3.14)	0.075
3	305	2.77(1.87–4.1)	1.10e−07	186	2.5(1.62–3.84)	1.60e−05
4	148	1.89(1.27–2.8)	0.0014	141	1.83(1.23–2.74)	0.0026
Stage T						
2	241	3.08(1.83–5.18)	8.10e−06	239	2.54(1.54–4.17)	0.00014
3	204	1.93(1.29–2.89)	0.0012	204	1.53(1.03–2.25)	0.032
4	38	2.25(0.88–5.8)	0.084	39	2.39(1.06–5.37)	0.03
Stage N						
0	74	4.39(1.01–19.1)	0.031	72	3.8(0.87–16.54)	0.056
1	225	3.07(1.94–4.88)	5.10e−07	222	2.79(1.77–4.38)	3.70e−06
2	121	2.74(1.71–4.37)	1.30e−05	125	3.17(1.75–5.76)	6.20e−05
3	76	2.56(1.44–4.56)	0.00093	76	2.2(1.24–3.92)	0.0059
1 + 2 + 3	422	2.38(1.79–3.16)	7.20e−10	423	2.4(1.72–3.35)	1.10e−07
Stage M						
0	444	2.36(1.74–3.19)	1.00e−08	443	2.46(1.72–3.5)	2.70e−07
1	56	3.15(1.67–5.93)	0.00021	56	1.72(0.93–3.18)	0.081
Lauren classification						
Intestinal	320	2.92(2.1–4.05)	2.50e−11	263	3.25(1.95–5.43)	1.90e−06
Diffuse	241	2.27(1.49–3.45)	7.90e−05	231	1.9(1.26–2.87)	0.0019
Differentiation						
Poor	165	1.81(1.06–3.08)	0.028	121	2.51(1.29–4.88)	0.0052
Moderate	67	1.65(0.75–3.63)	0.21	67	1.92(0.88–4.19)	0.096

### The potential association between NOTCH3 level and cancer patient prognosis

**Figure 3:**
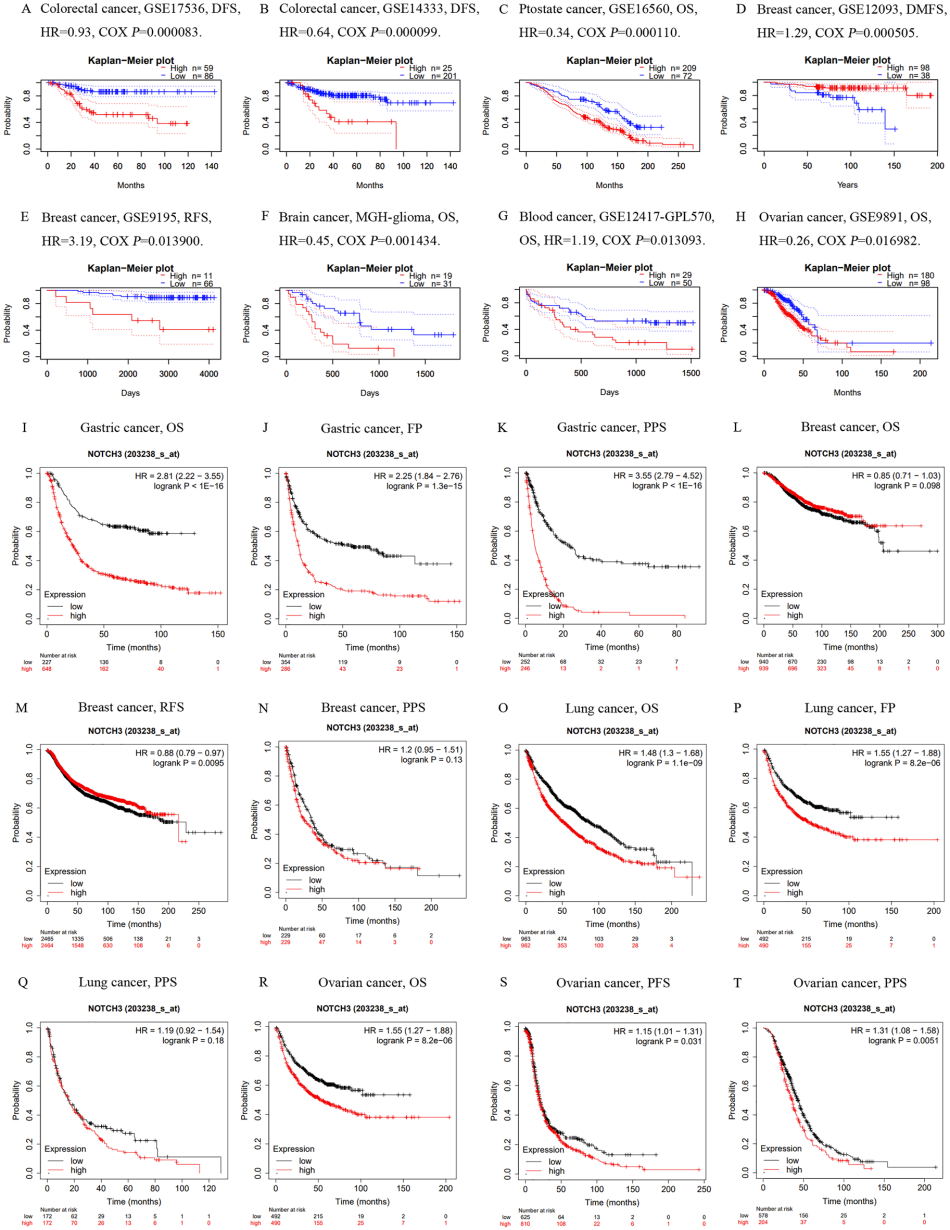
Correlation between NOTCH3 and prognosis of various types of cancer in the PrognoScan (**A**–**H**) Correlation between NOTCH3 and prognosis of various types of cancer in the Kaplan–Meier plotter database (**I**–**T**). OS, overall survival; PFS, progression-free survival; RFS, recurrence-free survival.

Using GEPIA2, clinical data from TCGA were utilized to investigate the predictive significance of NOTCH3 expression level in order to better determine the prognostic potential of NOTCH3 in different cancer types^22^. In colorectal cancer (DFS GSE = 17,536(177 sample)^23^, HR = 0.93, *P* = 0.000083; DFS GSE = 14,333(290 sample)^24^, HR = 0.64, *P* = 0.000099) and prostate cancer (OS GSE = 16,560(186 sample)^25^, HR = 0.34, *P* = 0.000110) (Fig. 3C), there was a marginally negative correlation found between high expression of NOTCH3 and a poor prognosis.Low NOTCH3 expression was marginally associated with a worse prognosis in one cohort (GSE12093, HR = 1.29, *P* = 0.000505) (136 samples)^26^, which included 136 samples at the DMFS stage of breast cancer. In contrast, high NOTCH3 expression was marginally associated with a worse prognosis in another cohort (GSE9195, HR = 3.19, *P* = 0.013900) (112 samples)^27,28^ that included 112 samples at the RFS stage of breast cancer. It’s interesting to note that NOTCH3 was also discovered to significantly affect patient prognoses for a few other non-gastrointestinal tumours. In brain cancer (OS MGH-glioma (136 sample)^27^, HR = 0.45, *P* = 0.001434), blood cancer (OS GSE = 12,417-GPL570(163 sample)^29,30^, HR = 1.19, *P* = 0.013093), and ovarian cancer (OS GSE = 9891(285 sample)^31^, HR = 0.26, *P* = 0.016982), high expression of NOTCH3 was likewise linked to a poor prognosis.

We then evaluated the relationship between NOTCH3 expression and prognosis in a variety of tumour types using the Kaplan–Meier plotter database^32^. The results showed that an upgrade was significantly associated with a poorer prognosis in the following tumour types: ovarian cancer (OS HR = 1.55, 95% CI = 1.27–1.88, *P* = 8.2e−06; PFS HR = 1.15, 95% CI = 1.01–1.31, *P* = 0.031; PPS HR = 1.31, 95% CI = 1.08–1.58, *P* = 0.0051), lung cancer (OS HR = 1.48, 95% CI = 1.3–1.63, *P* = 1.1e−09; FP HR = 1.55, 95% CI = 1.27–1.88, *P* = 8.2e−06), ovarian cancer (OS HR = 1.55, 95% CI = 1.27–1.88, *P* = 8.2e−06), lung cancer (OS HR = 1.48, 95% CI = 1.31–2.68, *P* = 1.1e−16), lung cancer (OS HR = 1.48, 95% CI = 1.27–1.88, *P* = 1.1e−16), and stomach cancer (OS HR = 1.45, 95% CI = 1.01–1. It’s interesting to note that in terms of the prognosis and survival connection of patients with breast cancer, the expression level of NOTCH3 in the Kaplan–Meier plotter database produced findings that were comparable to those in the GEPIA database (RFS HR = 0.88, 95% CI = 0.79–0.97, *P* = 0.0095). (Fig. 3I–T). NOTCH3 expression did not significantly correlate with the prognosis of patients with lung cancer (PPS HR = 1.19, 95% CI = 0.92–1.54, *P* = 0.18) or breast cancer (OS HR = 0.85, 95% CI = 0.61–2.03, *P* = 0.098; PPS HR = 1.2, 95% CI = 0.95–1.51, *P* = 0.13) (Fig. 3L,N,Q). In conclusion, it is evident that the poor prognosis in a variety of tumour types was strongly correlated with the high expression of NOTCH3.

### NOTCH3 expression correlated with immune infiltration levels in gastrointestinal cancer

**Figure 4:**
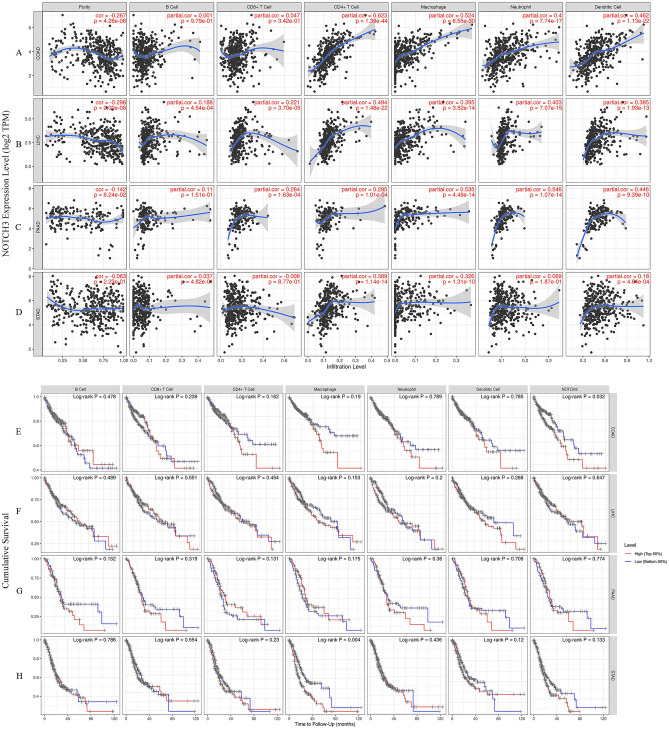
NOTCH3 expression is correlated with the level of immune infiltration in COAD, LIHC, PAAD and STAD. (**A**–**D**) NOTCH3 expression is correlated with the level of immune infiltration in COAD, LIHC, PAAD and STAD. (**E**–**H**) Kaplan–Meier plots of immune infiltration and NOTCH3 expression levels in COAD, LIHC, PAAD and STAD.

The frequency of lymphocyte infiltration into the tumour is an independent predictor of both survival and lymph node metastases in cancer patients. A few cancer types that can serve as prognostic indicators that are strongly correlated with the degree of immune infiltration are identified in the timer database^33^ in light of the relationship between NOTCH3 expression and immune infiltrating levels in different malignancies. When using genomic approaches to analyze immune infiltration in clinical tumour samples, tumour purity is a crucial issue to consider^34^. Consequently, we chose cancer types where the NOTCH3 expression level was substantially connected with the prognosis in GEPIA and considerably adversely correlated with tumour purity in TIMER^35^. There was a significant association between NOTCH3 levels and tumor purity (cor = − 0.267, *P* = 4.26e-08), CD4 + T cell (cor = − 0.623, *P* = 1.39e−44), macrophage (cor = − 0.524, *P* = 6.55e−30), neutrophil (cor = 0.4, *P* = − 7.74e−17) and dendritic cell infiltration (cor = − 0.462, *P* = 1.13e−22) in COAD (Fig. 4A). Similarly, positive correlations were identified with infiltrating levels of NOTCH3 (cor = − 0.296, *P* = 2.00e−08), B cell (cor = − 0.188, *P* = 4.54e−04), CD8 + T cell (cor = − 0.221, *P* = 3.70e−05), CD4 + T cell (cor = − 0.494, *P* = 1. 48e−22), macrophage (cor = − 0.395, *P* = 3.52e−14), neutrophil (cor = − 0.403, *P* = 7.07e−15) and dendritic cell (cor = − 0.385, *P* = 1.93e−13) infiltration in LIHC (Fig. 4B). A significant relationship was shown in the NOTCH3 expression and CD8 + T cell (cor = − 0.284, *P* = 1.63e−04), CD4 + T cell (cor = − 0.295, *P* = 1.01e−04), macrophage (cor = − 0.535, *P* = 4.49e−14), neutrophil (cor = − 0.546, *P* = 1.07e−14) and dendritic cell (cor = − 0.446, *P* = 9.39e−10) infiltration in PAAD (Fig. 4C). Interestingly, there was no significant correlation with tumor purity, B cell, CD8 + T cell, neutrophil and the expression of NOTCH3, despite the temporary relationship between CD4 + T cell (cor = − 0.389, *P* = 1.14e−14), macrophage (cor = − 0.326, *P* = 1.31e−10), dendritic cell (cor = − 0.18, *P* = 4.84e−04) infiltration and NOTCH3 levels in STAD. These results clearly show that NOTCH3 might attract immune cells to the tumour microenvironment (TME) in COAD, LIHC, PAAD, and STAD, particularly on dendritic cells, CD4 + T cells, and macrophages.

Further Kaplan–Meier plots were created using the TIMER database to investigate the connection between immune cell infiltration and NOTCH3 expression in COAD, LIHC, PAAD, and STAD. While no significant correlation was found between prognosis and immune cell infiltration or NOTCH3 expression (*P* > 0.05) in LIHC and PAAD (Fig. 4F,G), we did find that NOTCH3 expression (*P* = 0.032) significantly correlated with COAD prognosis and macrophage infiltration (*P* = 0.004) with STAD prognosis (Fig. 4E,H). These findings revealed that NOTCH3 was a potent regulator of immune cell infiltration in gastrointestinal cancer, with a potent influence on the infiltration of dendritic cells, CD4 + T cells, and macrophages.

### Correlations of NOTCH3 in immune marker expression

**Figure 5:**
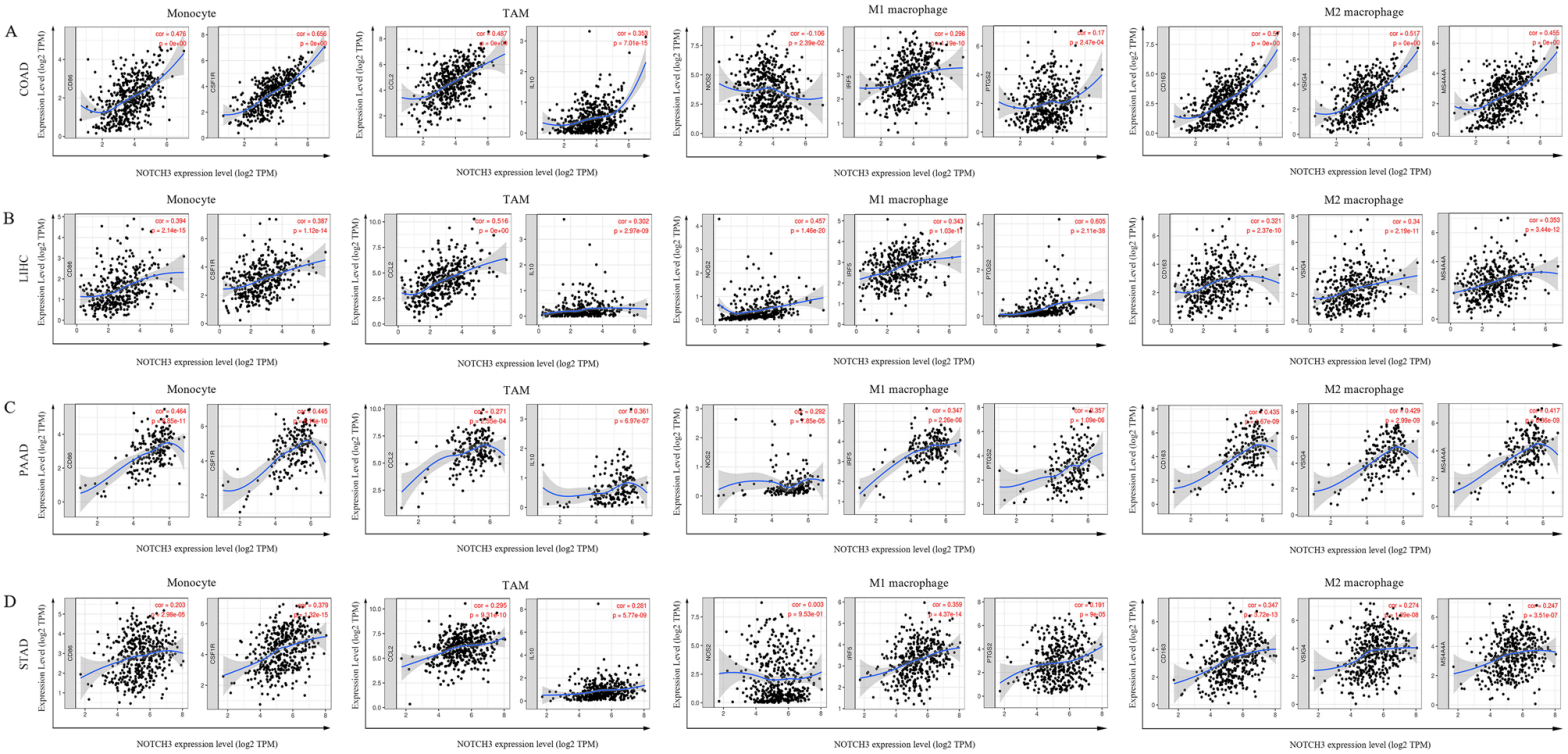
Correlation analysis between NOTCH3 expression and immunological marker set in COAD, LIHC, PAAD and STAD. (**A**) Scatterplots of correlations between NOTCH3 expression and gene markers of monocytes, TAMs, and M1 and M2 macrophages in COAD. (**B**) Scatterplots of correlations between NOTCH3 expression and gene markers of monocytes, TAMs, and M1 and M2 macrophages in LIHC. (**C**) Scatterplots of correlations between NOTCH3 expression and gene markers of monocytes, TAMs, and M1 and M2 macrophages in PAAD. (**D**) Scatterplots of correlations between NOTCH3 expression and gene markers of monocytes, TAMs, and M1 and M2 macrophages in STAD.

We thoroughly performed a correlation study between NOTCH3 and the representative marker genes of diverse immune infiltrating cells using the TIMER and GEPIA databases^36^ in order to further investigate the relationship between NOTCH3 expression and immune-infiltrating cell types (Fig. 5). In fact, we discovered that NOTCH3 expression in COAD, LIHC, PAAD, and STAD was substantially linked with the majority of gene markers for monocyte markers (CD86, CSF1R), TAM indicators (CCL2, IL10), M1 macrophage markers (INOS, IRF5, PTGS2), and M2 macrophage markers (CD163, VSIG4, MS4A4A). In other words, NOTCH3 expression and gene markers showed a substantial correlation (*P* < 0.05; Fig. 4), which was in line with the results found in the TIMER database (Table 3). According to the findings, NOTCH3 may be correlated with COAD, LIHC, PAAD, and STAD macrophage polarization.

**Table 3:** Correlation analysis between NOTCH3 and relate genes and markers of immune cells in TIMER.

Description	Gene markers	COAD				LIHC			
		None		purity		None		purity	
		Cor	*P*	Cor	*P*	Cor	*P*	Cor	*P*
CD8^+^T cell	CD8A	0.242	***	0.182	***	0.246	***	0.155	**
	CD8B	0.133	**	0.1	*	0.116	*	0.017	0.758
T cell (general)	CD3D	0.234	***	0.159	**	0.152	**	0.042	0.434
	CD3E	0.347	***	0.289	***	0.318	***	0.213	***
	CD2	0.271	***	0.2	***	0.276	***	0.172	**
B cell	CD19	0.277	***	0.21	***	0.178	***	0.09	*
	CD79A	0.363	***	0.3	***	0.22	***	0.097	*
Monocyte	CD86	0.476	***	0.434	***	0.394	***	0.31	***
	CD115(CSF1R)	0.656	***	0.64	***	0.387	***	0.29	***
TAM	CCL2	0.487	***	0.442	***	0.516	***	0.449	***
	CD68	0.487	***	0.449	***	0.275	***	0.174	**
	IL10	0.353	***	0.329	***	0.302	***	0.194	***
M1 Macro-phage	INOS(NOS2)	− 0.106	*	− 0.132	**	0.457	***	0.449	***
	IRF5	0.296	***	0.301	***	0.343	***	0.356	***
	COX2(PTGS2)	0.17	***	0.122	*	0.605	***	0.561	***
M2 Macro-phage	CD163	0.57	***	0.545	***	0.321	***	0.228	***
	VSIG4	0.517	***	0.476	***	0.34	***	0.25	***
	MS4A4A	0.455	***	0.414	***	0.353	***	0.266	***
Neutro-phils	CD66 b(CEACAM8)	− 0.145	**	− 0.131	**	0.015	0.778	− 0.017	0.747
	CD11b (ITGAM)	0.649	***	0.627	***	0.384	***	0.319	***
	CCR7	0.444	***	0.384	***	0.4	***	0.295	***
Natural killer cell	KIR2DL1	0.085	*	0.055	0.27	0.053	0.31	0.042	0.439
	KIR2DL3	0.095	*	0.09	*	0.134	**	0.092	*
	KIR2DL4	0.056	0.234	0.014	0.782	− 0.008	0.882	− 0.054	0.318
	KIR3DL1	0.15	**	0.128	*	0.135	**	0.131	*
	KIR3DL2	0.187	***	0.159	**	0.091	*	0.058	0.28
	KIR3DL3	− 0.026	0.582	− 0.026	0.604	0.086	*	0.08	0.137
	KIR2DS4	0.141	**	0.133	**	0.051	0.325	0.053	0.329
Dendritic cell	HLA-DPB1	0.489	***	0.444	***	0.359	***	0.259	***
	HLA-DQB1	0.24	***	0.185	***	0.241	***	0.137	*
	HLA-DRA	0.326	***	0.264	***	0.369	***	0.279	***
	HLA-DPA1	0.404	***	0.355	***	0.388	***	0.301	***
	BDCA-1(CD1C)	0.359	***	0.324	***	0.417	***	0.325	***
	BDCA-4(NRP1)	0.701	***	0.681	***	0.564	***	0.547	***
	CD11c(ITGAX)	0.642	***	0.618	***	0.453	***	0.384	***
Th1	T-bet(TBX21)	0.352	***	0.304	***	0.207	***	0.103	*
	STAT4	0.248	***	0.183	***	0.327	***	0.282	***
	STAT1	0.336	***	0.312	***	0.35	***	0.324	***
	IFN-γ(IFNG)	0.107	*	0.064	0.198	0.087	*	0.018	0.744
	TNF-α(TNF)	0.27	***	0.239	***	0.302	***	0.207	***
Th2	GATA3	0.512	***	0.484	***	0.434	***	0.372	***
	STAT6	0.263	***	0.252	***	0.347	***	0.362	***
	STAT5A	0.376	***	0.375	***	0.358	***	0.309	***
	IL13	0.216	***	0.192	***	0.103	*	0.087	0.106
Tfh	BCL6	0.53	***	0.495	***	0.185	***	0.191	***
	IL21	0.161	***	0.122	*	0.002	0.963	− 0.055	0.306
Th17	STAT3	0.356	***	0.333	***	0.457	***	0.433	***
	IL17A	− 0.133	**	− 0.125	*	0.13	*	0.136	*
Treg	FOXP3	0.6	***	0.571	***	0.246	***	0.202	***
	CCR8	0.548	***	0.529	***	0.479	***	0.438	***
	STAT5B	0.498	***	0.516	***	0.352	***	0.423	***
	TGFβ(TGFB1)	0.672	***	0.642	***	0.541	***	0.482	***
T cell exhaustion	PD-1(PDCD1)	0.368	***	0.307	***	0.219	***	0.117	*
	CTLA4	0.376	***	0.327	***	0.161	**	0.063	0.247
	LAG3	0.31	***	0.251	***	0.016	0.755	− 0.086	0.11
	TIM-3(HAVCR2)	0.484	***	0.451	***	0.407	***	0.331	***
	GZMB	0.086	*	0.066	0.186	0.061	0.239	− 0.026	0.625

Description	Gene markers	PAAD				STAD			
		None		purity		None		purity	
		Cor	*P*	Cor	*P*	Cor	*P*	Cor	*P*
CD8^+^T cell	CD8A	0.241	**	0.196	**	0.132	**	0.121	0.0188
	CD8B	0.198	**	0.147	0.0545	0.087	0.0769	0.091	0.0783
T cell(general)	CD3D	0.225	**	0.183	*	0.04	0.419	0.03	0.565
	CD3E	0.269	***	0.228	**	0.121	*	0.119	*
	CD2	0.254	***	0.208	**	0.101	*	0.098	*
B cell	CD19	0.237	**	0.206	**	0.203	***	0.212	***
	CD79A	0.238	**	0.193	*	0.147	**	0.14	**
Monocyte	CD86	0.464	***	0.429	***	0.203	***	0.199	***
	CD115(CSF1R)	0.445	***	0.397	***	0.379	***	0.372	***
TAM	CCL2	0.271	***	0.239	**	0.295	***	0.289	***
	CD68	0.428	***	0.388	***	0.194	***	0.182	***
	IL10	0.361	***	0.327	***	0.281	***	0.28	***
M1 Macro-phage	INOS(NOS2)	0.292	***	0.31	***	0.003	0.953	0.009	0.864
	IRF5	0.347	***	0.352	***	0.359	***	0.355	***
	COX2(PTGS2)	0.357	***	0.357	***	0.191	***	0.179	***
M2 Macro-phage	CD163	0.435	***	0.384	***	0.347	***	0.337	***
	VSIG4	0.429	***	0.372	***	0.274	***	0.277	***
	MS4A4A	0.417	***	0.373	***	0.247	***	0.237	***
Neutrophils	CD66 b(CEACAM8)	0.107	0.155	0.077	0.319	0.022	0.655	0.031	0.544
	CD11b(ITGAM)	0.479	***	0.442	***	0.383	***	0.386	***
	CCR7	0.255	***	0.222	**	0.271	***	0.286	***
Natural killer cell	KIR2DL1	0.103	0.171	0.113	0.141	0.052	0.289	0.052	0.311
	KIR2DL3	0.119	0.113	0.109	0.158	− 0.012	0.806	− 0.021	0.69
	KIR2DL4	0.004	0.961	0.005	0.947	− 0.095	*	− 0.111	*
	KIR3DL1	0.025	0.735	0.001	0.988	0.049	0.317	0.04	0.436
	KIR3DL2	0.185	*	0.1554	*	0.042	0.398	0.051	0.32
	KIR3DL3	0.06	0.422	0.03	0.696	− 0.098	*	− 0.096	*
	KIR2DS4	0.008	0.915	0.02	0.794	0.009	0.861	− 0.012	0.814
Dendritic cell	HLA-DPB1	0.333	***	0.296	***	0.115	*	0.113	*
	HLA-DQB1	0.194	**	0.149	*	0.071	0.151	0.061	0.288
	HLA-DRA	0.337	***	0.299	***	0.03	0.546	0.027	0.605
	HLA-DPA1	0.319	***	0.282	***	0.06	0.223	0.058	0.263
	BDCA-1(CD1C)	0.222	**	0.198	**	0.21	***	0.216	***
	BDCA-4(NRP1)	0.536	***	0.504	***	0.574	***	0.557	***
	CD11c(ITGAX)	0.528	***	0.5	***	0.372	***	0.377	***
Th1	T-bet(TBX21)	0.164	*	0.139	*	0.188	***	0.199	***
	STAT4	0.165	*	0.179	*	0.217	***	0.217	***
	STAT1	0.368	***	0.338	***	0.189	***	0.189	***
	IFN-γ(IFNG)	0.155	*	0.127	*	− 0.071	0.147	− 0.069	0.178
	TNF-α(TNF)	0.284	***	0.264	***	0.174	***	0.178	***
Th2	GATA3	0.383	***	0.38	***	0.232	***	0.237	***
	STAT6	0.212	**	0.212	**	0.305	***	0.316	***
	STAT5A	0.352	***	0.334	***	0.377	***	0.37	***
	IL13	0.135	*	0.142	*	0.1	*	0.132	*
Tfh	BCL6	0.438	***	0.426	***	0.499	***	0.489	***
	IL21	0.134	*	0.136	*	0.008	0.872	0.012	0.813
Th17	STAT3	0.474	***	0.451	***	0.504	***	0.497	***
	IL17A	− 0.038	0.612	− 0.061	0.431	− 0.12	*	− 0.123	*
Treg	FOXP3	0.435	***	0.414	***	0.302	***	0.309	***
	CCR8	0.439	***	0.411	***	0.331	***	0.33	***
	STAT5B	0.362	***	0.417	***	0.547	***	0.549	***
	TGFβ(TGFB1)	0.647	***	0.647	***	0.602	***	0.602	***
T cell ex-haustion	PD-1(PDCD1)	0.295	***	0.261	***	0.19	***	0.203	***
	CTLA4	0.338	***	0.3	***	0.151	**	0.157	**
	LAG3	0.15	*	0.127	*	0.077	0.116	0.073	0.157
	TIM-3(HAVCR2)	0.491	***	0.456	***	0.259	***	0.255	***
	GZMB	0.19	*	0.141	*	− 0.015	0.763	− 0.03	0.555

Then, utilizing the TIMER and GEPIA databases, we investigated the relationship between NOTCH3 and immune cell infiltration levels based on sets of immunological markers in COAD, LIHC, PAAD, and STAD. A more precise comparison was made between NOTCH3 expression and the quantities of cell-specifically produced protein molecules in CD8 + T cells, total T cells, B cells, monocytes, TAMs, M1 and M2 macrophages, neutrophils, NK cells, DCs, Th1 cells, Th2 cells, Tfh cells, Th17 cells, Tregs, and exhausted T cells. The findings demonstrated a positive and statistically significant correlation between the expression of NOTCH3 and the following: monocyte markers (CD86, CD115), TAM markers (CCL2, CD68, IL10), M1 macrophage markers (INOS, IRF5, COX2), M2 macrophage markers (CD163, VSIG4, MS4A4A), neutrophil markers (CD11b, CD66b, CCR7), DC markers (HLA-DPB1, HLA-DQB1, HLA-DRA, HLA-DPA1, BDCA-1, BDCA-4, CD11c), Th1 markers (T-bet, STAT4, STAT1, TNF-α), Th2 markers (GATA3, STAT6, STAT5A, IL13), Tfh markers (BCL6), Th17 markers (STAT3), Treg markers (FOXP3, CCR8, STAT5B, TGFβ1), and T cell exhaustion (PD-1, TIM-3) in COAD, LIHC, PAAD, and STAD. Monocyte, TAM, M2 Macrophage, Neutrophils, Dendritic Cell, Th2, Tfh, Treg, and T cell exhaustion macrophage indicators in COAD, LIHC, PAAD, and STAD are linked with NOTCH3 expression. Consequently, these findings unequivocally demonstrate that NOTCH3 is positively linked with immune invading cells in COAD, LIHC, PAAD, and STAD, indicating that NOTCH3 is crucial for immune escape in gastrointestinal cancer.

### Upregulation of COX-2 and NLRP3 with activated NF-κB signaling in HGC-27 cell line

**Figure 6:**
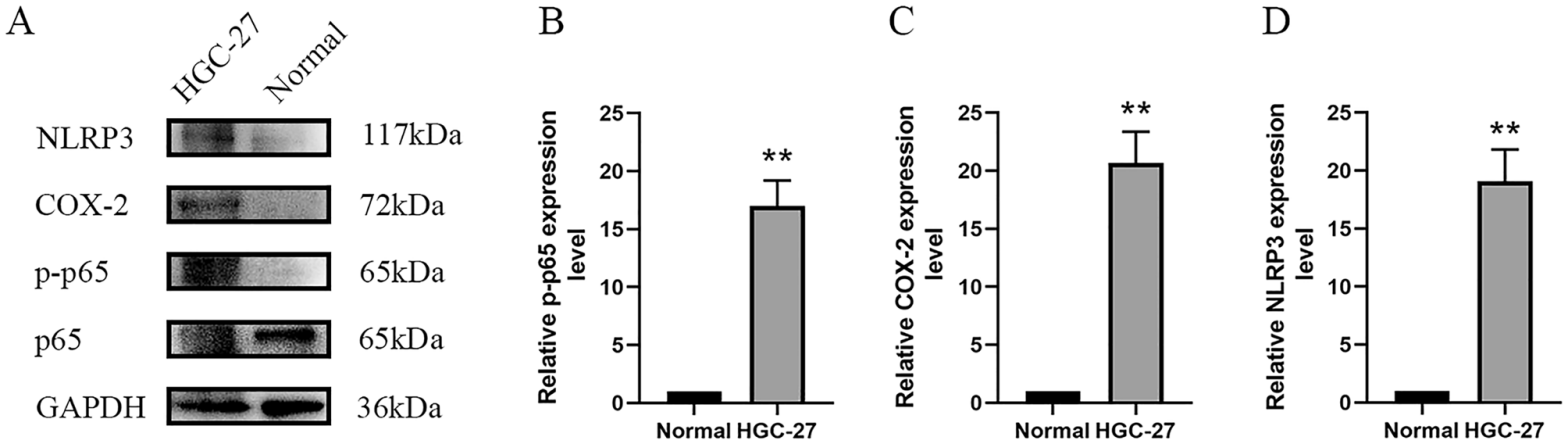
Differential expression of NLRP3, COX-2, and NF-κB p-p65 in HGC-27 cell line and normal gastric tissue. (**A**) The expression levels of NLRP3, COX-2 and p-p65 in HGC-27 cell line and normal gastric tissue were determined by western blot analysis. (**B**) The relative quantification of bands was performed by optical density scanning of (**A**). COX stands for cyclooxygenase. NLRP3 represents NOD-like receptor protein 3, and p-p65 represents phosphorylated nuclear factor kappa-light-chain-enhancer of activated B cells p65 subunit.

NLRP3, COX-2, and NF-κB p-p65 are highly expressed in the HGC-27 gastric cancer cell line. In previous qPCR and Western blot experiments, we found that NOTCH3 expression was abnormally elevated in HGC-27 cells (Fig. 1). To investigate the expression trends of NOTCH3 and immune infiltration-related factors in gastric cancer cells, we examined the protein expression of NLRP3, COX-2, and NF-κB p-p65 in HGC-27 cell line and normal gastric tissue. Compared to normal gastric tissue, the protein expression of NLRP3, COX-2, and p-p65 in HGC-27 cells was significantly increased (*P* < 0.01; Fig. 6). Thus, the expression of NLRP3, COX-2, and NF-κB p-p65 in GC cells positively correlates with NOTCH3 expression, suggesting a positive regulatory relationship between NOTCH3 expression and immune infiltration in GC cells.

## Discussion

NOTCH3, a signal receptor, which controls cell fate synergistically acts with other NOTCH proteins to participate in the regulation of various tissue stem cells and the plasticity of vascular smooth muscle phenotype in vascular remodeling^37^. NOTCH has a significant effect in differentiation, its dysregulation leads to multiple malignant tumors^38^. Ellisen^39^ reported that the translocation t (7; 9) in T cell acute lymphoblastic leukemia (T-ALL) can lead to a fusion of genes encoding the β chain of T cell receptors and the TAN1/NOTCH1 gene, which was the first evidence to prove the tumorigenic potential of NOTCH. NOTCH3 is a novel target protein of the prolyl-isomerase Pin1. It can regulate the processing of NOTCH3 protein and stabilize the cleaved product, which leads to the high expression of the intracellular domain (N3IC), ultimately enhancing Notch3-dependent invasiveness properties^40^.

In this report, we assessed the expression level of NOTCH3 as it connected to the prognosis of various cancers according to the independent Oncomine and GEPIA databases, revealing clear differences between tumor and normal tissue expression of NOTCH3 in different cancers. The result of Oncomine database shows that NOTCH3 is overexpressed in lots of cancers such as bladder, colorectal, gastric, head and neck, kidney, leukemia, liver, lung, lymphoma, ovarian and pancreatic cancers compared to normal tissues (Fig. 2A). The similar results are also available in the TCGA database. The expression of NOTCH3 is much higher in in BLCA, CHOL, COAD, ESCA, HNSC, KIRC, LIHC, LUAD, LUSC, READ, STAD, THCA and UCEC (Fig. 2B). However, NOTCH3 is under-expressed in some esophageal, head and neck, kidney, ovarian and sarcoma cancers, and such results seem to be contradictory. The difference in NOTCH3 expression levels of different cancer types may be related to the data collection methods and potential biological characteristics. In any case, based on these results, we found that NOTCH3 is abnormally highly expressed in gastrointestinal tumors (COAD, LIHC, PAAD, STAD), breast cancer and lung cancer, and increased NOTCH3 expression was associated with poor prognosis. Kaplan–Meier Plotter and timer database also showed a high NOTCH3 expression correlated with poor prognosis (Figs. 3). Further research found that overactive NOTCH3 was closely related to poor prognosis, gender, stage, T stage, N stage, M stage, Lauren classification and differentiation in gastrointestinal cancer (Table 1). These results indicate that NOTCH3 may have the potential as a prognostic biomarker for gastrointestinal tumors.

Our research had a more important discovery, revealing that NOTCH3 expression was related to the degree of immune infiltration. We found that the expression of NOTCH3 was moderately positively correlated with the degree of macrophage infiltration, CD4 +, neutrophil infiltration and DC, and slightly positively correlated with the degree of CD8 + (Fig. 4A). Then correlation analysis between immune cell infiltration and gastrointestinal tumor prognosis was tested. We found that the correlation between the expression of certain immune marker genes strongly suggested that in gastrointestinal tumors, NOTCH3 can control the infiltration and interaction of immune cells in the tumor microenvironment. In addition, there was a moderately correlation between NOTCH3 and monocyte, TAM, M1/M2 macrophage markers (including CD86, CSF1R, CCL2, IL10, IRF5, PTGS2, IRF5, CD163, VSIG4 and MS4A4A) (Fig. 5).

We further found NOTCH3 levels in gastrointestinal tumor to correlate with markers of Dendritic cell(HLA-DPB1, HLA-DRA, HLA-DPA1, BDCA-4), Tfh(T-bet, STAT4, STAT1, TNF-α), Treg cells (FOXP3, CCR8, STAT5B, TGFβ) and T cell exhaustion (PD-1, CTLA4, LAG3, TIM-3) (Table 2). This indicated that NOTCH3 played a role in promote Treg response and inhibit T cell-mediated immunity. Through Western blot and qPCR experiments, we assessed the expression of NOTCH3 in gastric cancer cells, revealing a significant increase in both gene transcription and protein levels within the HGC-27 cell line (Fig. 1). Further Western blot analysis confirmed notable elevations in protein expression levels associated with immune infiltration in HGC-27 cells. Specifically, we observed significant increases in the expression levels of NLRP3 and COX-2 proteins, along with activation of NF-κB phosphorylation (Fig. 6). This finding further supports our earlier database findings regarding the potential association between NOTCH3 expression and immune cell infiltration in gastrointestinal tumor patients. Cui^41^ analyzed pathological specimens of 48 gastric cancer patients and found that with lower infiltration of activated CD8 + T cells and higher infiltration of immunosuppressive cells including Tregs and M2 macrophages in the tumor microenvironment.

Despite the strengths of our study in identifying NOTCH3 as a prognostic biomarker and elucidating its correlation with immune infiltration in gastrointestinal cancers, several limitations must be acknowledged.Firstly, our findings primarily rely on data obtained from public databases such as Oncomine, TIMER, PrognoScan, and GEPIA. While these databases provide a robust and extensive resource for gene expression and clinical outcome data, they also come with inherent limitations. The data quality and consistency can vary, potentially introducing biases and affecting the reliability of our results.Secondly, although we performed experimental validations using Western blot and qPCR to confirm the expression of NOTCH3, these validations were limited in scope and scale. Further experimental studies involving larger sample sizes and additional techniques such as immunohistochemistry (IHC) and flow cytometry would provide more definitive evidence and strengthen our conclusions.Additionally, the correlation analyses between NOTCH3 expression and immune infiltration markers were conducted using in silico tools. Although these tools are powerful for hypothesis generation and preliminary analysis, they cannot fully capture the complex interactions within the tumor microenvironment. Experimental validation through techniques such as IHC and flow cytometry would provide more definitive evidence and strengthen our conclusions.Lastly, the clinical data used to assess the prognostic significance of NOTCH3 were retrospective in nature. Prospective clinical trials with larger, more diverse patient cohorts are necessary to confirm the clinical utility of NOTCH3 as a prognostic biomarker.In conclusion, while our study provides valuable insights into the potential role of NOTCH3 in gastrointestinal cancers, these limitations highlight the need for further validation and experimental studies to fully elucidate its clinical relevance and underlying mechanisms.

Taken together, these results highlight the ability of NOTCH3 to potentially regulate immune cell recruitment and activation in gastrointestinal tumor. In summary, NOTCH3 may be an important regulator of immune cell infiltration in patients with gastrointestinal tumors, a valuable prognostic biomarker and even a potential therapeutic target. Consistent with the far-reaching impact that Notch has on development and homeostasis, aberrant activity of the pathway is also linked to the initiation and progression of several malignancies, and NOTCH can in fact be either oncogenic or tumor suppressive depending on the tissue and cellular context.

### Supplementary Information

Supplementary Figure 1.

Supplementary Information 2.

Supplementary Information 3.

## Data Availability

The data in this article all comes from the TCGA database. The datasets generated and analysed during the current study are available in the following public repositories. The datasets generated and analysed during the current study are available in the Ocomine database (http://www.oncomine.org) with filter conditions set to: Fold change > 1.5, *P*-value < 0.001, and gene ranking overall. The datasets generated and analysed during the current study are available in the PrognoScan database (http://www.abren.net/PrognoScan), with a Cox *P*-value < 0.05 used to recalculate the cutoff. The datasets generated and analysed during the current study are available in the Kaplan–Meier Plotter database (http://kmplot.com/analysis/), with the log-rank P-value and the hazard ratio (HR) with 95% confidence intervals calculated. The datasets generated and analysed during the current study are available in the TIMER database (https://cistrome.shinyapps.io/timer/), which includes 10,897 samples of 32 cancer types from the Cancer Genome Atlas (TCGA). The datasets generated and analysed during the current study are available in the GEPIA database (http://gepia.cancer-pku.cn/index.html), which analyzes RNA-seq data from 9736 tumor samples and 8587 normal control samples from the TCGA and GTEx data sets. All data generated or analysed during this study are included in this published article and its supplementary information files. If you have any further questions or require additional information, please contact the corresponding author.
