# Supplementary figures and images for: NOTCH3 as a prognostic biomarker and its correlation with immune infiltration in gastrointestinal cancers
Source: Sci Rep. 2024 Jun 21;14:14327. doi: 10.1038/s41598-024-65036-x (PMC11192884; doi:10.1038/s41598-024-65036-x)

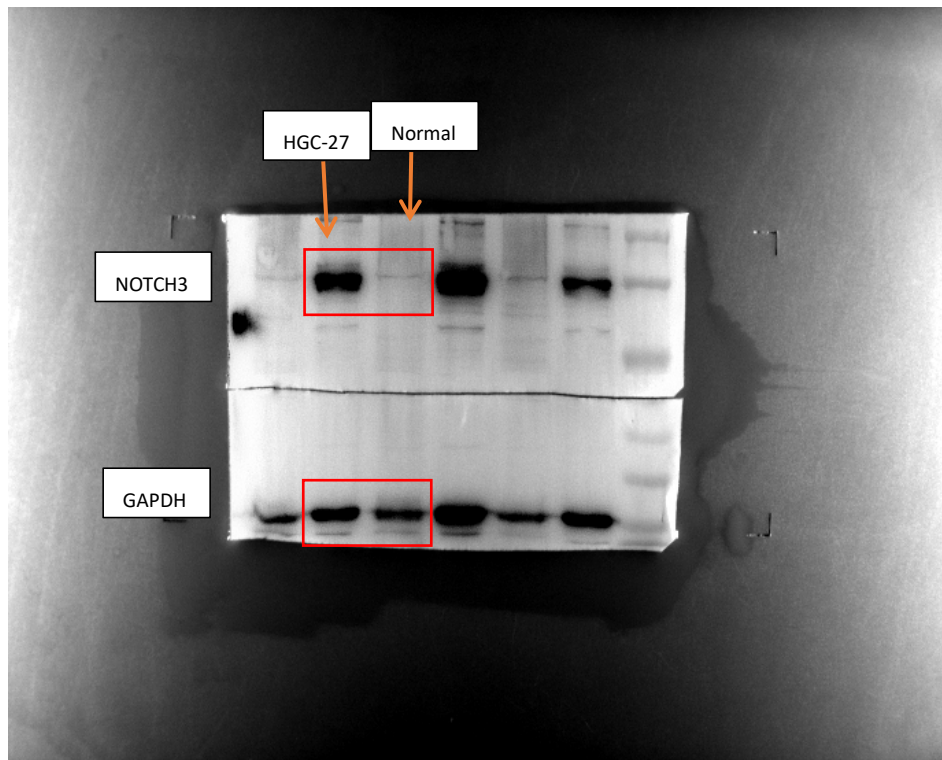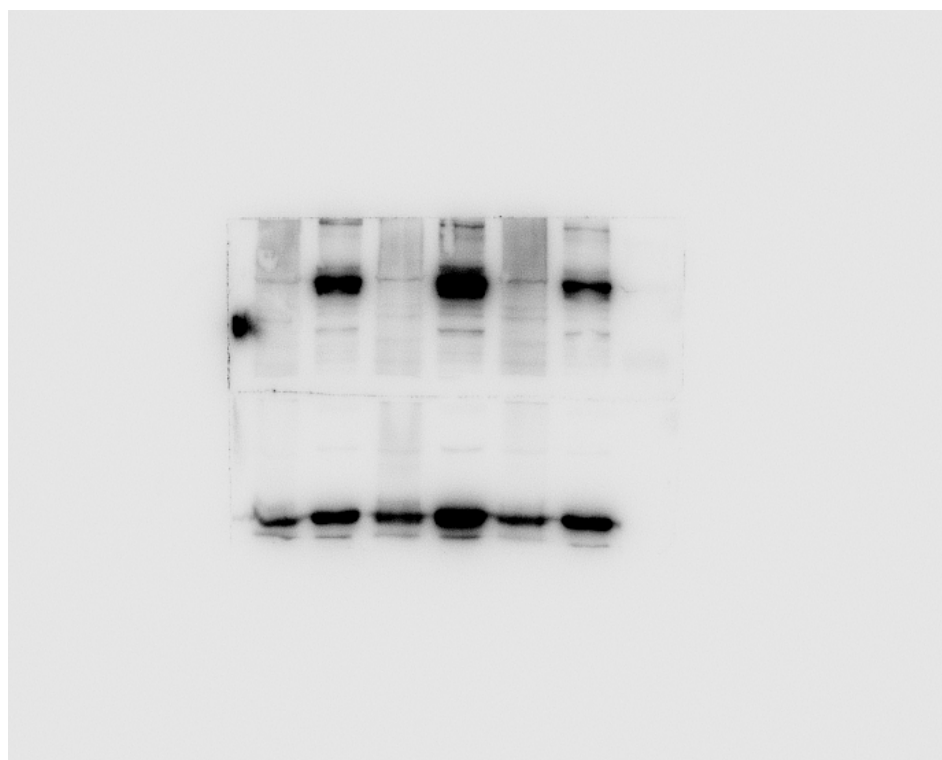

Supplement: Supplementary file 1 — Supplementary Figure 1. [file 41598_2024_65036_MOESM1_ESM.pdf]

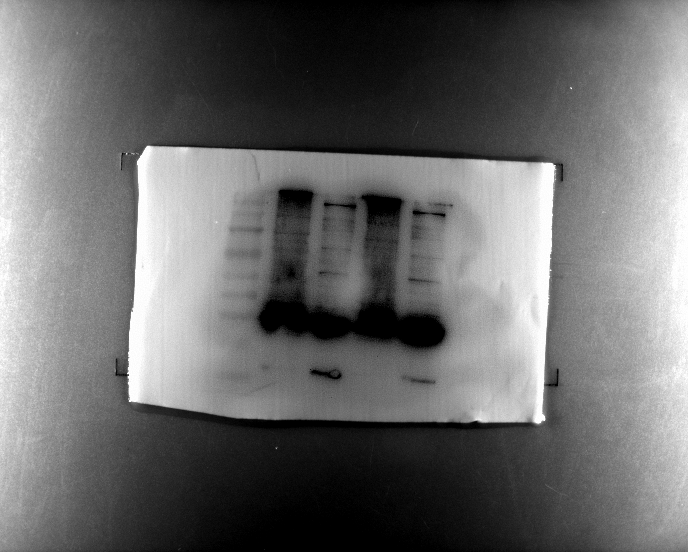

Supplement: Supplementary file 2 — Supplementary Information 2. [file 41598_2024_65036_MOESM2_ESM.zip › ╒√─ñ/HGC27║═╬╕╫Θ╓»-20s╢■╒┼╒√─ñMerge.tif]

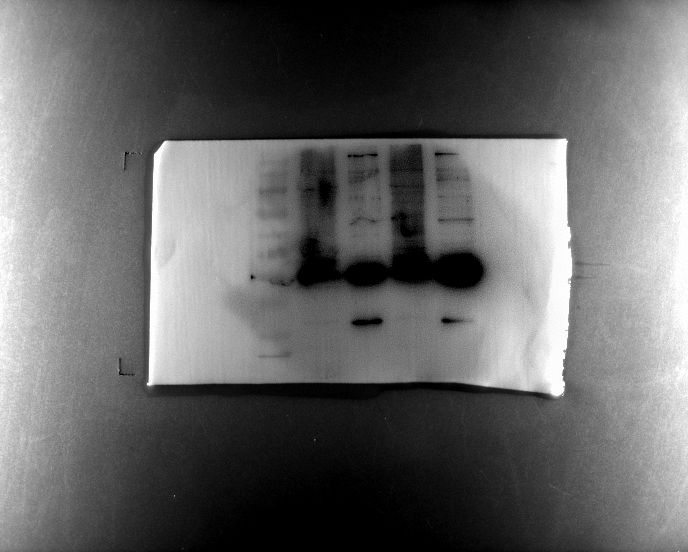

Supplement: Supplementary file 2 — Supplementary Information 2. [file 41598_2024_65036_MOESM2_ESM.zip › ╒√─ñ/HGC27║═╬╕╫Θ╓»-20s╚2╒┼╒√─ñMerge.tif]

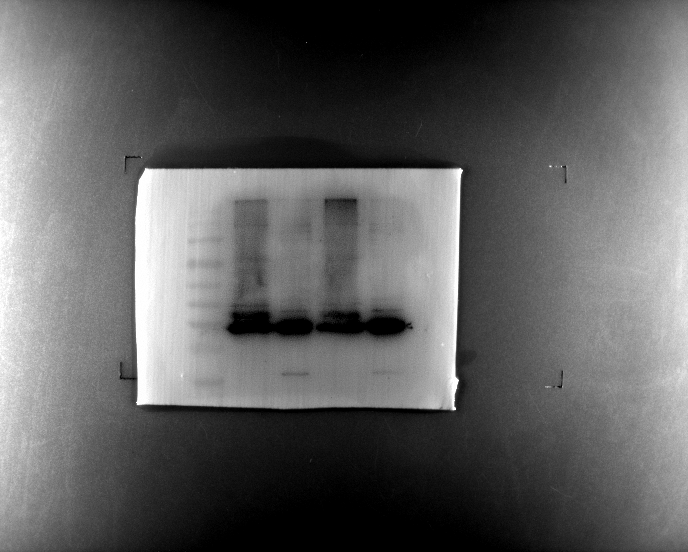

Supplement: Supplementary file 2 — Supplementary Information 2. [file 41598_2024_65036_MOESM2_ESM.zip › ╒√─ñ/HGC27║═╬╕╫Θ╓»-20s╥╗╒┼╒√─ñMerge.tif]
